# Auricular Acupuncture to Lower Blood Pressure Involves the Adrenal Gland in Spontaneously Hypertensive Rats

**DOI:** 10.1155/2020/3720184

**Published:** 2020-11-20

**Authors:** Huong Thi Mai Nguyen, Der-Yen Lee, Hung-Ming Wu, Ching-Liang Hsieh

**Affiliations:** ^1^International Master Program in Acupuncture, College of Chinese Medicine, China Medical University, Taichung 40402, Taiwan; ^2^Graduate Institute of Integrated Medicine, College of Chinese Medicine, China Medical University, Taichung 40402, Taiwan; ^3^Department of Neurology, Changhua Christian Hospital, Changhua 500, Taiwan; ^4^Graduate Institute of Acupuncture Science, College of Chinese Medicine, China Medical University, Taichung 40402, Taiwan; ^5^Chinese Medicine Research Center, China Medical University, Taichung 40402, Taiwan; ^6^Department of Chinese Medicine, China Medical University Hospital, Taichung 40447, Taiwan

## Abstract

Auricular acupuncture is used to treat cardiac-related diseases such as hypertension. Therefore, the purpose of the present study was to investigate the effects of auricular acupuncture on blood pressure (BP) in spontaneously hypertensive rats (SHRs). The treatment group (TG) received auricular electroacupuncture (EA) at the auricle heart (CO15) and auricle shenmen (TEF3) points. Heart rate (HR) and BP, GABA-A expression, catecholamine, and neurotransmitter levels were measured. The HR was reduced after 7 auricular EA treatments compared with controls (all *p* < 0.05). Systolic BP and diastolic BP also decreased immediately and throughout the treatments compared with controls (all *p* < 0.05). The reduction of BP and HR was reversed by bicuculline injection 30 min before auricular EA treatment (all *p* < 0.05). GABA levels in the adrenal gland were higher with auricular EA treatment compared with the control group at 4 h (*p* < 0.05). Levels of serum noradrenaline and adrenaline were reduced at 15 min after final auricular EA treatment compared with the normal control group (both *p* < 0.05). The lowering of BP and HR by auricular EA is possibly mediated via vagal afferents from the concha to the nucleus of the solitary tract. After signal integration in the medulla oblongata, it may be transmitted through sympathetic efferent or vagal efferent or through multiple signaling pathways simultaneously to the atrionector of heart and the adrenal medulla. Further study is warranted.

## 1. Introduction

Hypertension is defined by the mean values of 2 or more blood pressure (BP) measurements in which diastolic pressure (DBP) is higher than or equal to 90 mmHg and systolic BP (SBP) is equal to or higher than 140 mmHg [1]. Hypertension can be classified according to its etiology into essential (primary) hypertension, where no cause is identified in 95% of the hypertension population and secondary hypertension, which has an identifiable cause [2].

The sympathetic nervous system can influence cardiac output and peripheral vascular resistance to modulate blood pressure. Increased sympathetic activity can cause hyperkinetic circulation and peripheral vasoconstriction, resulting in the generation of hypertension [3]. Hypertension is frequently associated with sympathetic overactivity, which has harmful effects on target organs and is also a predictive factor for cardiovascular complications. In addition, autonomic nervous system abnormalities can induce, for example, tachycardia and obesity and is also a risk factor for cardiovascular disorders [4]. Hypertension is due to a disarray of sympathetic and parasympathetic cardiovascular regulation; increased sympathetic nerve activity or reduced vagal cardiac tone causes hypertension [5]. Therefore, reducing sympathetic nerve activity or increasing parasympathetic activity can be targeted for controlling hypertension.

Adrenaline acts as a neurotransmitter and can enhance the generation of hypertension. Adrenaline can be released by sympathetic nerve endings associated with noradrenaline [6]. Renal sympathetic nerves are linked with the sympathetic nervous system and the control of long-term arterial pressure. Chronic increases in renal adrenergic activity can induce increases of arterial pressure, and chronic decreases in this activity can induce decreases in arterial pressure [7]. One study reported that the adrenal medulla is innerved by sympathetic and parasympathetic efferents and afferents [8]. Noradrenaline is an efficacious vasoconstrictor hormone, whereas adrenaline is less efficacious. Sympathetic nerve endings cause the adrenal gland to secrete both noradrenaline and adrenaline into the blood stream. Other neurotransmitters in the adrenal gland such as *γ*-aminobutyric acid (GABA) [9] and serotonin [10] also contribute to the regulation of BP, through catecholamine secretion from adrenal chromaffin cells. One study demonstrated that young people with high BP had higher serum cortisol levels compared with controls [11]. Clearly, sympathetic nerve activity and the adrenal gland, including the cortex and medulla, have a close relationship to hypertension.

Auricle conchae are innervated by the auricular branches of the vagus nerve. Acupuncture at a concha can induce afferent projection through the vagus nerve to the nucleus of solitary tract (NTS). Neurons that synapse in the NTS join in autonomic reflex and the regulation of autonomic function. Auricle heart points (CO15), located in the conchae, and CO15 and auricle *shenmen* points (TEF3) have been used to treat hypertension [12, 13]. Our previous study shows that 2 Hz electroacupuncture (EA) at bilateral Zusanli (ST36) and Shangjuxu (ST37) increases high frequency component of heart rate variability (HRV), whereas 50 Hz EA increases low frequency component of HRV, therefore, suggesting 2 Hz EA can increase vagal activity, while 50 Hz increases sympathetic activity [14]. A study finds 2 Hz EA significantly inhibit cardiovascular press reflex, but 40 Hz or 100 Hz cannot in the same points stimulation [15]. Therefore, the purpose of the present study was to investigate the effect and mechanism of EA at points CO15 and TEF3 in lowering BP in SHRs using 2 Hz EA.

## 2. Methods

### 2.1. Animals

Male SHRs aged 5 to 7 weeks were purchased from BioLASCO Taiwan Co., Ltd. The rats were raised in the animal center of China Medical University (CMU) with a 12 h light/dark cycle. The room temperature and humidity were controlled by an air conditioner at 20–24 °C and 50%–70%, respectively. The rats were free-fed and provided sufficient drinking water. Animal use was approved by the Institutional Animal Care and Use Committee of CMU and followed the recommendations in the *Guide for the Use of Laboratory Animals* (National Academy Press).

### 2.2. Experimental Procedure

The SHR started at the 9 weeks of age, with SBP greater than 140 mmHg and DBP greater than 90 mmHg. Therefore, the experiments were started at the 10^th^ week of age in the present study.

### 2.3. Experiment 1: To Investigate the Effect of Auricular Acupuncture on Heart Rate (HR) and BP in SHRs

#### 2.3.1. Groups

A total of 18 SHRs were randomly divided into 3 groups (*n* = 6/group) as follows: (1) a control group (CG), where the rats were received no auricular EA treatment; (2) a sham group (SG), for which 2 Hz EA was applied to both auricle helix 1 (Hx1) and helix 2 (Hx2) points; and (3) a treatment group (TG), where the procedure was identical to that of the SG, but 2 Hz EA was applied to the CO15 and TEF3 points.

### 2.4. Measurement of HR and BP in SHRs

The HR and BP of the rat tails were measured under 2% isoflurane gas anesthesia by using a tail-cuff apparatus (LE 5002, Panlab Harvard). Measurements of HR and BP were made before and after auricular EA treatment, which was delivered between 8 : 00 a.m. and 12 : 00 noon. Before the measurements, the rats were maintained at 28°C for 10 min with a heat pad, and the pulsations of the tail arteries could be detected. The values of BP and HR in the present study are the average of three measurements.

### 2.5. Acupuncture Treatment

Stainless acupuncture needles (No. 32 G, Quian-Hui, New Taipei City, Taiwan; 0.27 mm in diameter, 13 mm in length) were inserted into either the CO15 and TEF3 (TG) or the Hx1 and Hx2 (SG) points. CO15 is located in the middle of the deepest part of the concha. TEF3 is located at the tip of the fossa triangularis between the upper and lower legs of the antihelix. Hx1 is located on the helix, inferior to the tubercle of the helix, and Hx2 is inferior to Hx1 [12, 13].

The ipsilateral CO15 and TEF3 were a pair, as were Hx1 and Hx2. Both pairs were connected to an electrostimulation apparatus (Trio 300, Japan). The frequency of the stimulus was 2 Hz. The intensity of the stimulus was a slightly visible twitch, and the treatment duration was 20 min. The SHRs received the treatment 3 times per week for 3 continual weeks, that is, at 10, 11, and 12 weeks of age. The rats were sacrificed under 5% isoflurane deep anesthesia at 4 h after the final treatment was completed. Blood from the heart, brain tissue, and adrenal glands of the rats were collected.

### 2.6. Adrenalectomy

The rats were anesthetized under 2% isoflurane gas. A dorsal incision from 2 cm lateral to the vertebral column and from the first to third lumbar vertebrae of the SHR was made, and the dorsal muscle was dissected and pulled away until the adrenal gland was exposed. The bilateral adrenal gland with its surrounding adipose tissue was removed.

### 2.7. Experiment 2: To Confirm Auricular EA Lowering of BP Involves GABA of Adrenal Gland in SHRs

At the 10^th^ week of age, 18 male SHRs were randomly divided into 3 groups of 6 as follows: a normal group (NG), to which no auricular EA was applied; an EA group (EG), where the rats received 2 Hz of EA at CO15 and TEF3 points; and a bicuculline group (BG), in which the rats received 2 Hz EA at CO15 and TEF3 points as well as bicuculline (an antagonist of GABA-A; 1 mg/kg dissolved in 10% dimethyl sulfoxide (DMSO) 99.5% + 10% Tween 20 + 80% distilled water or deionized water-DDW) was slowly injected into the tail 30 min prior to EA treatment. Auricular EA treatment was applied 3 times/week for 3 consecutive weeks, that is, at 10, 11, and 12 weeks of age. Both HR and BP were measured before and after the third, sixth, and ninth treatment. The rats were sacrificed under 5% isoflurane gas anesthesia at 15 min after the final treatment.

### 2.8. Liquid Chromatography-Electrospray Ionization-Mass Spectrometry Analysis

Prior to analysis, 50 *μ*L of serum or adrenal gland water extract was completely mixed with 200 *μ*L of methanol through vigorous vortexing. The samples were centrifuged at 14 000 rpm for 10 min, and 150 *μ*L of supernatant was collected for drying at 35°C for 2 h in a vacuum concentrator. The dried samples were dissolved in 50 *μ*L of ultrapure water to measure the levels of dopamine and cortisol by monitoring the m/z transition 154 to 137 and 363.2 to 121.06, respectively, through ES + MRM mode using an Xevo TQ-XS (Waters).

Other neurotransmitters were measured using a VION-IMS Q-Tof after a dansylation process. The liquid chromatography-electrospray ionization-mass spectrometry (LC-EM-MS) analysis was performed as previously described [16] to detect catecholamine and neurotransmitter intensity in the adrenal gland and serum. For metabolite dansylation, 20 *μ*L of 20 mg/mL dansyl chloride in 50 mM NaHCO3, pH 9.5 was added to dissolve the samples. The mixture was incubated at 70°C for 2 h, then 80 *μ*L of ultrapure water was added, and the mixture incubated at 70°C for 30 min. After centrifugation at 14 000 rpm for 10 min, the supernatant of the reaction mixture was transferred into an insert vial.

The flow rate was set at 0.2 mL/min with a column temperature of 35°C. Separation was performed using reversed-phase LC on a BEH C18 column (2.1 × 100 mm, Walters) with a 7.5 *μ*L sample injection. The elution was started from 99% mobile phase A (ultrapure water + 0.1% formic acid) and 99% mobile phase B (100% methanol + 0.1% formic acid), held at 1% B for 0.5 min, raised to 90% B at 5.5 min, held at 90% B for 1 min, and then lowered to 1% B and held at 1 min. The column was equilibrated by pumping 1% B for 4 min. An LC-ESI-MS chromatogram was acquired using ESI + mode as follows: 2.5 kV capillary voltage, 100 °C source temperature, desolvation temperature of 250°C, maintained cone gas at 10 L/h and desolvation gas at 600 L/h, and acquisition by the MS^E^ mode from 100 to 1000 m/z and a 0.5 s scan time. Data were analyzed using UNIFI software (Waters) with an illustrated chromatogram and summarized in an integrated area of signals. The results are presented as a ratio compared with control rats.

### 2.9. Data Preparation and Statistical Analysis

All data are reported as means ± standard error. SPSS software was used to analyze the data. Significant differences were analyzed using one-way analysis of variance followed by Tukey's test (post hoc) and then mixed models analyses to see how auricular EA and sham EA affected the change of HR and BP over longitudinal treatment. The statistical significance was accepted at *p* < 0.05.

## 3. Results

### 3.1. Effect of Auricular EA on HR and BP in SHRs

**Figure 1:**
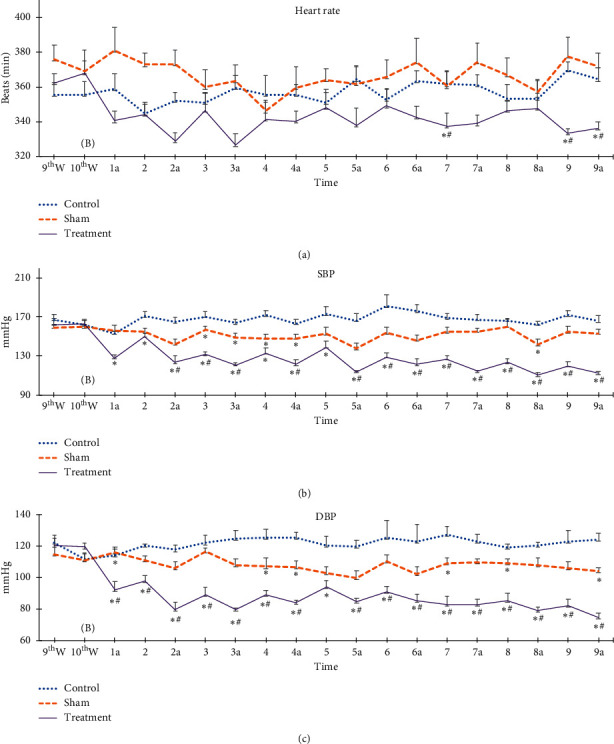
Effect of auricular electroacupuncture (EA) on heart rate (HR) and blood pressure (BP). 9^th^W: ninth week from birth; 10^th^W: tenth week from birth; (B): baseline prior to auricular EA treatment; 2–9 times: measurement of HR was before auricular EA; 1a–9a times: measurement of HR was after auricular EA. Control: control group without auricular EA; Sham: sham group, auricular EA at bilateral auricle helix 1 and helix 2 points; Treatment: treatment group, auricular EA at bilateral auricle heart and *shenmen* points; SBP: systolic blood pressure; DBP: diastolic blood pressure; one-way ANOVA, Tukey post hoc test, ^*∗*^*p* < 0.05 versus control, ^#^*p* < 0.05 versus sham.

HR was not significantly different at baseline (before first auricular EA treatment) among the CG, SG, and TG (Figure 1(a)). HR was not significantly different between the SG and CG throughout 9 treatments, whereas HR was lower in the TG than in the CG and SG after the seventh and before and after the ninth auricular EA treatment (Figure 1(a)). These results demonstrated that auricular EA at CO15 and TEF3 after 7 treatments could reduce HR in SHRs.

SBP was also not significantly different at baseline among the groups (Figure 1(b)). SBP was lower in the SG than in the CG before the third and fourth treatment and after the second, third, fourth, and eighth treatment (Figure 1(b)), whereas, at other treatment times, no significant difference between the CG and SG was observed (Figure 1(b)); SBP was lower in the TG than in the CG and SG before and after every auricular EA treatment (Figure 1(b)). No significant difference between the SG and TG after the first or before the second, fourth, or fifth treatment (Figure 1(b)) was identified.

DBP also did not differ at baseline (Figure 1(c)). DBP was lower in the SG than in the CG after the first, fourth, and ninth and before the fourth, seventh, and eighth treatment (Figure 1(c)); otherwise, no significant difference existed (Figure 1(c)). DBP was lower in the TG than in the CG and SG before and after every treatment (Figure 1(c)), except that it did not significantly differ between the SG and TG before the fifth treatment (Figure 1(c)). To summarize the results, auricular EA at CO15 and TEF3 could immediately lower SBP and DBP, whereas these effects for auricular EA at Hx1 and Hx2 were weak.

The interaction between treatment in the SG or in the TG and times of treatment was not significant in HR analysis; however, the interaction between treatment and times of treatment was significant in BP including SBP and DBP analysis (Table 1).

### 3.2. Effect of Bicuculline with Auricular EA on HR and BP in SHRs

**Figure 2:**
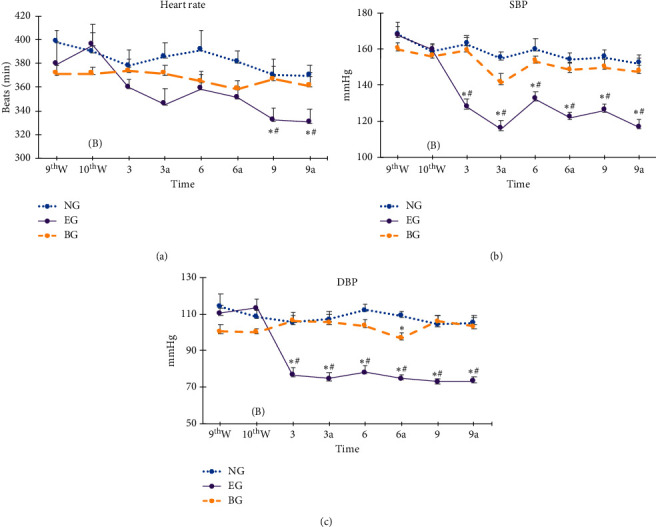
Effect of bicuculline on auricular electroacupuncture (EA) at bilateral auricle heart and shenmen points in lowering heart rate (HR) and blood pressure (BP). 9thW: ninth week from birth; 10thW: tenth week from birth; (B): Baseline, measurement of HR and BP prior to auricular EA; 3–9 times: measurement of HR and BP before auricular EA; 3a–9a: measurement of HR and BP after auricular EA; SBP: systolic blood pressure; DBP: diastolic blood pressure. One-way ANOVA, Tukey post hoc test, ^*∗*^*p* < 0.05 versus NG, ^#^*p* < 0.05 versus BG; NG: normal control without auricular EA treatment; EG: group with auricular EA at bilateral auricle heart and shenmen acupoints; BG: group with bicuculline injection 30 min prior to auricular EA.

HR was lower before and after the ninth treatment in the EG than in the NG and BG (Figure 2(a)). HR was not significantly different before or after the third and sixth treatment among the NG, EG, and BG (Figure 2(a)).

SBP in the EG was lower than in the NG and BG before and after the third, sixth, and ninth treatment (Figure 2(b)), whereas SBP did not significantly differ between the NG and BG before or after the third, sixth, or ninth auricular EA treatment (Figure 2(b)).

DBP in the EG was lower than in the NG and BG before and after the third, sixth, and ninth treatment (Figure 2(c)). SBP did not significantly differ between the NG and BG before or after the third, sixth, or ninth treatment, with the exception of before the sixth treatment, when DBP was lower in the BG than in the NG (Figure 2(c)). Taken together, the results demonstrated that the effectiveness of auricular EA in reducing HR and lowering SBP and DBP could be reversed by prior bicuculline injection.

### 3.3. Effect of Auricular Acupuncture on Neurotransmitters in the Adrenal Gland and Serum at 4 Hours after the Final Auricular EA

**Figure 3:**
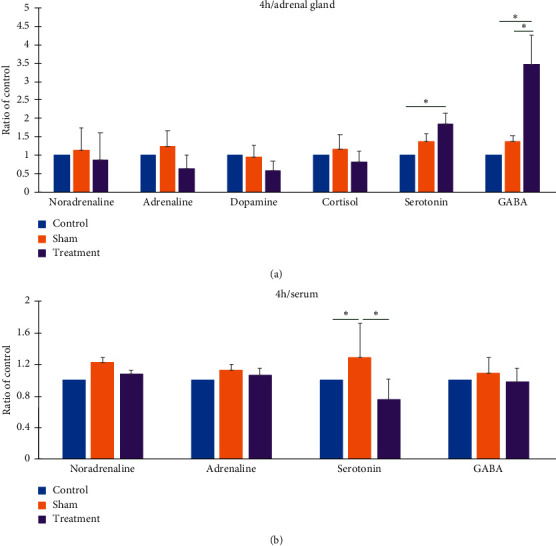
Effect of auricular electroacupuncture (EA) on catecholamine, cortisone, and neurotransmitter levels in the adrenal gland (a) and in serum (b) at 4 h after final auricular EA treatment. Control: control group without auricular EA treatment; Sham: sham group, auricular EA at bilateral auricle helix 1 and helix 2; Treatment: treatment group, auricular EA at bilateral auricle heart and shenmen points; Ratio of control: the value compared to the value of the control; ^*∗*^*p* < 0.05, one-way ANOVA, Tukey post hoc test.

The noradrenaline, adrenaline, dopamine, and cortisone levels of the adrenal gland were not significantly different at 4 h after the final auricular EA treatment among the groups (Figure 3(a)). Although the serotonin levels were higher in the TG than in the CG (Figure 3(a)), they were not significantly different between CG and SG or between the SG and TG (Figure 3(a)). The GABA levels of the adrenal gland were higher in the TG than in the CG and SG, whereas the levels were not significantly different between the CG and SG (Figure 3(a)) at 4 h after the final treatment.

The noradrenaline, adrenaline, serotonin, and GABA levels of serum were not significantly different among the groups at 4 h after treatment (Figure 3(b)). Taken together, the results suggest that auricular EA at CO15 and TEF3 could increase the serotonin and GABA levels of the adrenal gland, whereas auricular EA at Hx1 and Hx2 could not produce a similar effect.

### 3.4. Effect of Auricular Acupuncture on Neurotransmitters in the Adrenal Gland and Serum at 15 Minutes after the Final Auricular EA

**Figure 4:**
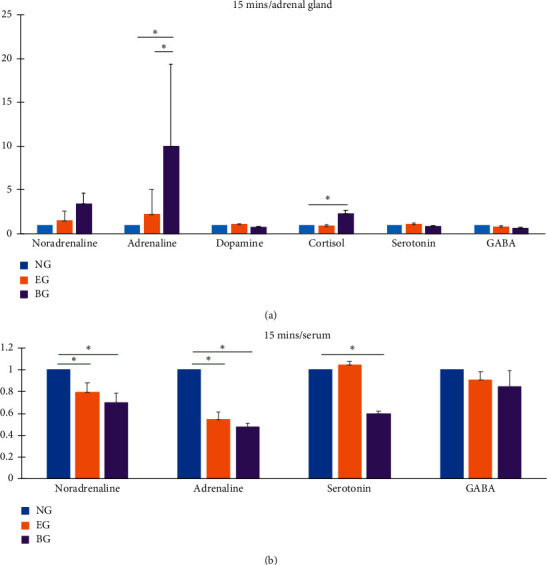
Effect of auricular electroacupuncture (EA) on catecholamine and neurotransmitter levels in the adrenal gland (a) and in serum (b) at 15 min after final auricular EA treatment. NG: normal control group without auricular EA treatment; EG: EA group receiving auricular EA at auricle heart and *shenmen* points; BG: bicuculline group with bicuculline injection 30 min prior to auricular EA treatment; Ratio of control: the value compared to the value of the control; ^*∗*^*p* < 0.05, one-way ANOVA, Tukey post hoc test.

The adrenaline levels of adrenal gland were higher in the BG than in either the NG or the EG (Figure 4(a)). The levels did not significantly differ between the NG and EG or between the EG and BG (Figure 4(a)). The cortisol levels of the adrenal gland were higher in the BG than in the NG (Figure 4(a)), whereas they were not significantly different between the NG and EG or between the EG and BG (Figure 4(a)). The levels of noradrenaline, dopamine, serotonin, and GABA in the adrenal gland did not significantly differ among the groups (Figure 4(a)) at 15 min after final auricular EA treatment.

The noradrenaline and adrenaline levels of serum were higher in the NG than in the EG and BG (Figure 4(b)). Their levels in the EG and BG were not significantly different (Figure 4(b)). The serotonin levels of serum were higher in the NG than in the EG (Figure 4(b)), whereas they were not significantly different between the NG and EG or between the EG and BG (Figure 4(b)). The serum GABA levels exhibited no significant difference among the groups (Figure 4(b)). The dopamine levels of serum were also not significantly different (Figure 4(b)). Taken together, the results indicate that the noradrenaline and adrenaline levels of serum at 15 min after final treatment were reduced by auricular EA at CO15 and TEF3, and this reduction could not be reversed by bicuculline pretreatment. In addition, bicuculline pretreatment could reduce the serum levels of serotonin.

## 4. Discussion

The results indicated that HR was reduced after 7 auricular EA treatments in the TG compared with the CG. This was not observed in the SG, suggesting auricular EA at CO15 and TEF3 could reduce HR but at Hx1 and Hx2 it could not. In addition, the results in this study also indicated that the interaction between treatment and times of treatment was not significant in HR analysis. EA at CO15 can induce signals through the auricular branch of the vagus nerve to the NTS. These signals then retransmit from the dorsal motor nucleus (DMN) and nucleus ambiguus via the vagal efferent pathway to the sinoatrial node of the heart to reduce HR. These signals can also involve interneurons passing through the caudal and rostral ventrolateral medullae and then through the intermediolateral cell column of the spinal cord to modulate sympathetic nerve activity in order to influence the sinoatrial node of the heart, reducing HR [17]. Taken together, the results suggest that auricular EA at CO15 and TEF3 could increase vagal efferents and modulate sympathetic nerve activity, affecting the sinoatrial node and reducing HR.

Noradrenaline and adrenaline are the main catecholamines of the adrenal gland; sympathetic fibers from preganglionic fibers and then via the sympathetic ganglion extend to chromaffin cells off the adrenal medulla to secrete adrenaline and noradrenaline, which can increase HR and BP [18]. Although the innervation of the parasympathetic nerve to the adrenal gland is an ongoing debate, recent reports of observations of vagus fibers in adrenal glands [19, 20] suggest that stimulating the vagus nerve can alter adrenal gland hormones through a vagal efferent connection, which suggests another pathway for an auricular EA treatment effect on the glands.

Our results also indicated that SBP was lower in the SG only before and after the third and fourth treatment and after the second and eighth compared with the CG. DBP was reduced only before the fourth, seventh, and eighth treatment and after the first, fourth, and ninth treatment in the SG compared with the CG. Both SBP and DBP were lower in the TG than in the CG and SG before and after every treatment except before the fifth treatment, when the SBP and DBP in the TG were similar in the SG. These results suggest auricular EA at CO15 and TEF could lower BP and the interaction between treatment and times of treatment was significant. In addition, our results indicated that GABA levels in the adrenal gland were increased at 4 h after the celiac plexus final auricular EA treatment in the TG. Several studies have demonstrated that the vagus nerve can alter adrenal gland hormones through a vagal efferent connection [19, 20]. The neural interaction between the NTS and dorsal motor nucleus of the vagus links signals from vagal afferents to efferents. The vagus nerve efferent fibers from the DMN of the vagus also innervate ganglia in the, which is the likely origin of these postganglionic catecholaminergic fibers [21]. The adrenal glands also are innervated by celiac ganglia, which contains preganglionic sympathetic fibers [22]. Several studies have reported that orally or intravenously administrated GABA could lower BP in both animals and humans [23, 24]. Oral administration of GABA significantly decreases the SPB in SHRs but not in Wistar-Kyoto rats (normal tensive rats) [25]. The ingestion of milk containing GABA can reduce BP in patients with mild hypertension [26]. GABA has been identified in various endocrine organs, and it is synthesized and stored by steroid-producing cells of the adrenal cortex. GABA functions in a paracrine manner to modulate the release of catecholamines from the adrenal medulla via GABA-A receptors. Matsuoka et al. proved that GABA has an inhibitory effect on a trans-synaptic-evoked increase in excitability in adrenal medulla cells. They suggested that GABA-A receptor stimulation diminishes the total amount of catecholamine secretion [27]. Harada et al. determined that GABA has a dual effect in regulating the secretion of catecholamine in that it enhances its release at low levels and diminishes it at high levels [9]. In addition, in the adrenal medulla, GABA is synthesized in adrenal chromaffin cells, stored in chromaffin granules, and released in response to nicotinic acetylcholine receptor activation [27], and the vagus nerve innervates the adrenal medulla [28]. Our results also indicated that serum noradrenaline and adrenaline levels were lower in the EG (with auricular EA at CO15 and TEF3) and BG compared with the NG at 15 min after final treatment, but GABA levels of the adrenal gland increase in TG (with auricular EA at CO15 and TEF3) at 4h after final treatment. Therefore, the decrease of noradrenaline and adrenaline in the serum is independent of the use of GABA inhibitors, and GABA levels in the adrenal gland. The increase of GABA levels in the adrenal gland possible is a delay effect of auricular EA at CO15 and TEF3. GABA is gamma-aminobutyric acid, an important inhibitory neurotransmitter involved in a variety of metabolic activities. GABA can act on the vascular movement center of the spinal cord, effectively promote vascular dilatation, and achieve the purpose of lowering blood pressure [29]. Studies have found that GABA can also inhibit the secretion of antidiuretic hormone and effectively promote the dilation of blood vessels, so as to reduce BP [30]. Other experts found that GABA and its metabolites have a strong inhibitory effect on the activity of angiotensin converting enzyme [31–33]. This suggests that GABA produces antihypertensive effects not only by inhibiting the release of catecholamine from the adrenal medulla, but also by other means.

The vagus nerve innervates aortic baroreceptors. The stimulation of the vagus nerve causes a vasodilation of the veins and arterioles throughout the peripheral circulatory system and decrease HR and the strength of heart contraction. Therefore, a baroreceptor response in the artery reflex causes a decrease of arterial pressure through decreasing peripheral resistance and cardiac output [34]. Article acupoints Hx1 and Hx2 are distributed by the great auricular nerve, which is a superficial branch of the cervical nerve plexus and communicates with the auricular branch of the vagus nerve through the posterior branch; [35] therefore, the lowering of BP by auricular EA may also partly involve the baroreceptor reflex.

Our results indicated that the serotonin levels of the adrenal gland were higher in the TG compared with the CG at 4 h after final treatment. Yokoyama et al. reported that intracellular Ca^2+^ responses do not represent changes in adrenal chromaffin cells, whereas serotonin can reduce an acetylcholine-induced Ca^2+^ response in these cells [36]. Serotonin inhibits nicotine-induced currents and catecholamine release in cultured bovine adrenal chromaffin cells [10, 37], suggesting the serotonin levels of the adrenal gland can inhibit acetylcholine-induced excitability in adrenal cells and reduce catecholamine release.

The results also indicated that pretreatment with bicuculline can revise auricular EA at CO15 and TEF3 by lowering BP and can reduce HR in SHRs. These results are in agreement with the studies of Wible et al., which used bicuculline [1.0 mg/kg intravenous (IV)] [38], and Hsu et al., which used 3 mg/kg IP [39]; both reported an increase of mean BP. Barman and Gebber determined that bicuculline (1.5 mg/kg IV) can completely block the inhibition of reflex vagal bradycardia induced by brachial plexus afferent stimulation, and 2.5 mg/kg IV was the maximal blockade for hypothalamic stimulation [40]. Humphrey and McCall reported that bicuculline at 0.25 to 1.0 mg/kg IV can attenuate the vagal sympathetic inhibitory response and increase the locking of sympathetic nerve discharge to the arterial pulse [41]. To sum up, bicuculline pretreatment can reverse auricular EA–induced BP lowering, and the reduced HR possibly results from bicuculline's inhibition of vagal nerve activity and sympathetic nerve discharge.

The results demonstrated that levels of both serum noradrenaline and adrenaline were lower in the EG (with auricular EA at CO15 and TEF3) compared with the NG (without any treatment) at 15 min after final treatment but not at 4 h. Catecholamine secretion from the adrenal gland into circulation is rapid. The half-time of disappearance is approximately 2.5 min under resting conditions, and low levels of catecholamine are released into the blood from the adrenal medulla and sympathetic nerve terminals. Under specific stress conditions, plasma adrenaline levels reach peak values 40-fold greater than in control undisturbed rats at approximately 20 min, and then the levels decline to approximately one-third of the peak levels, while noradrenaline levels are increased by approximately 6-fold [42]. Taken together, these findings indicate that GABA-A receptors are main pathways contributing to lowering BP through auricular EA.

## 5. Conclusion

The results of the present study indicated that HR was reduced after 7 auricular EA treatments at CO15 and TEF3, and SBP and DBP were decreased immediately in SHRs. The interaction between treatment and times of treatment was significant in the lower BP and that could be revised by bicuculline pretreatment. The GABA-A levels of the adrenal gland were increased at 4 h, and serum noradrenaline and adrenaline levels were reduced at 15 min after final treatment with auricular EA at CO15 and TEF3 suggesting the decrease of noradrenaline and adrenaline in the serum is independent of the use of GABA inhibitors and GABA levels in the adrenal gland. The adrenal gland is innervated by the vagus efferent nerve from DMN and the sympathetic postganglion fibers from the aortic renal ganglion. In this study, it cannot be proved that the stimulation of vagal efferent by auricular EA can increase GABA expression in adrenal gland, unless the effect of auricular EA on GABA disappears after cutting off the vagus nerve directly innervating the adrenal gland. Undoubtedly, the role of auricular EA in reducing BP is to transmit signals to the NTS through the vagus afferent nerve. After signal integration in the medulla oblongata, it may be transmitted through sympathetic efferent or vagal efferent or through multiple signaling pathways simultaneously to the atrionector of heart and the adrenal medulla. The signal efferent pathways are very complicated, so we should not draw a conclusion easily.

There are some shortcomings in the present study: (1) the NG, EG, and BG were only tested for 15 minutes, and the CG, TG, and SG were tested for only 4 hours after final auricular EA treatment; complete study is need in the future; (2) the study lack cutting off of vagus nerve directly innervating the adrenal gland to investigate the changes of GABA levels. Further study is warranted.

## Figures and Tables

**Table 1:** Impact of auricular electroacupuncture (EA) on the change of heart rate (HR) and blood pressure (BP) over time.

Source	HR		SBP		DBP	
	*F*	*p*	*F*	*p*	*F*	*p*
Intercept	65647.822	0.0001	35775.553	0.0001	25248.255	0.0001
Treatment	75.420	0.0001	242.811	0.0001	240.786	0.0001
Times	1.207	0.258	9.267	0.0001	7.621	0.0001
Treatment *∗* times	1.171	0.289	3.971	0.0001	4.116	0.0001

Treatment with 2 values as sham group (SG) with auricular EA at bilateral auricle helix 1 and helix 2 points) and treatment group (TG) with auricular EA at bilateral auricle heart and *shenmen* acupoints; times with 19 values as 9^th^W: ninth week from birth; 10^th^W: tenth week from birth or baseline prior to auricular EA treatment; 2–9 times: measurement of HR was before auricular EA; 1a–9a times: measurement of HR was after auricular EA; F: F value; *p*: *p* value; SBP: systolic blood pressure; DBP: diastolic blood pressure; mixed models linear analysis.

## Data Availability

The data used to support the findings of this study are available from the corresponding author upon request.
